# Design of an AI-driven secure 5G-SDN framework with federated reinforcement learning for anomaly detection, mitigation, and attack forensics

**DOI:** 10.3389/frai.2026.1701944

**Published:** 2026-02-10

**Authors:** R. Shameli, Sujatha Rajkumar

**Affiliations:** School of Electronics Engineering, Vellore Institute of Technology, Vellore, India

**Keywords:** 5G SDN security, anomaly detection, blockchain security, efficient, federated reinforcement learning, spiking neural networks, transformer networks

## Abstract

**Introduction:**

The increasing adoption of Software-Defined Networking (SDN) in 5G networks has revolutionized network management. However, this paradigm shift has introduced critical security vulnerabilities, including data-plane anomalies, control-layer intrusions, and Distributed Denial-of-Service (DDoS) attacks. Existing intrusion detection approaches based on Convolutional Neural Networks (CNNs) and Long Short-Term Memory (LSTM) networks suffer from high computational overhead, long detection latency, and limited scalability, making them unsuitable for real-time 5G-SDN environments.

**Methods:**

This article proposes a novel multi-layered security framework for 5G-SDN that integrates EfficientNet with Knowledge Distillation (KD), Transformer Networks, Spiking Neural Networks (SNNs), Federated Reinforcement Learning (FRL), and blockchain technology. EfficientNet-KD enables lightweight and accurate anomaly detection at the data-plane layer. Transformer networks capture long-range temporal dependencies to enhance control-layer attack detection. SNNs are employed for ultra-low-latency attack classification by mimicking human brain neural processing. FRL supports decentralized and privacy-preserving mitigation across SDN controllers, improving scalability, while blockchain technology ensures the integrity and immutability of attack logs for forensic reliability.

**Results:**

The proposed framework was evaluated using multiple benchmark datasets, including CICIDS2017, UNSW-NB15, IoT-23, and InSDN. Experimental results demonstrate an average detection accuracy of 97.75%, detection latency of 15 ms, and less than 5% throughput degradation. Each detection consumes only 0.25 J of energy, achieving a 40% reduction in energy usage compared to traditional CNN- and LSTM-based approaches.

**Discussion:**

The results verify that the proposed framework provides a scalable, energy-efficient, and low-latency intrusion detection and mitigation solution for 5G-SDN environments. By integrating lightweight deep learning, neuromorphic computing, decentralized learning, and blockchain-based security, the framework effectively addresses the limitations of existing methods and offers a robust approach for securing next-generation 5G-SDN networks.

## 1 Introduction

At the basis of today, the integration of Software-Defined Networking (SDN) within the 5G networks has significantly transformed modern communication infrastructures by introducing programmability, flexibility, a centralized control process, and dynamic resource orchestration. SDN introduces centralized network intelligence, which separates the control plane from the data plane, enabling centralized policy enforcement and fine-grained traffic engineering across various network slices, while northbound APIs enable applications to define network behavior, southbound interfaces (e.g., OpenFlow) are usually used to handle communication between the controller and the data plane. Architectural change is needed for 5G infrastructures to support ultra-reliable low-latency communications, massive machine-type communications, and enhanced mobile broadband. Despite these benefits, 5G SDN networks increase the attack surface. The rapid evolution of 5G networks, along with a dependence on Software-Defined Networking (SDN), has provided enormous high-potency security threats. Old network security mechanisms designed for static architectures cannot be accepted for holding such dynamically programmable SDN environments, where threats evolve rapidly across data, control, and application layers. SDN-enabled 5G systems are dynamic and multi-layered, making static or monolithic security solutions inefficient for real-time reaction, scalability, and energy efficiency. However, this paradigm shift opened up the 5G networks to a new range of cyber threats (Wang et al., 2023; Zhao et al., 2024; Alsalem and Bhargava, 2024). Centralized control logic, configurable forwarding rules, and inter-controller communication allow data-plane irregularities, control-plane invasions, Distributed Denial-of-Service (DDoS) attacks, and policy manipulation.

Traditional security mechanisms (Zhang and Wang, 2024; Wang W. et al., 2024; Wang Y. et al., 2024) designed for conventional networks are inefficient due to the dynamic and adaptive nature of SDN-based architectures; as such, intelligent, real-time, and scalable threat detection and mitigation measures are required. Existing security frameworks are largely reliant on deep learning based intrusion detection systems (IDS) like Convolutional Neural Networks (CNNs) and long short-term memory (LSTM) networks to carry out anomaly detection in SDN environments. Conversely, these models demonstrate serious drawbacks in terms of computability, latency of inference, and adaptability to rapidly changing environments. Centralized security mechanisms are a bottleneck with a single point of failure for the network, through an increased risk of losing its resilience against profound cyber threats such as botnet-driven DDoS attacks, controller hijacking, and flow rule manipulation. A lack of tamper-proof attack forensic evidence further erodes the processes of accountability and recovery after any compromise. The already limited attack mitigation mechanisms further include the very shared characteristics of poor real-time responsiveness and lack of collaborative powers among SDN controllers, thus draining the very essence of the next-gen networks under such sinister threats. Deep learning-based intrusion detection, reinforcement learning-driven mitigation, and blockchain-assisted trust mechanisms have been studied to address these concerns. Most approaches divide detection, categorization, mitigation, and forensic logging. These systems often have high computational cost, delayed mitigation actions, or centralized training paradigms that compromise privacy and introduce single points of failure. Little effort has been made to align learning models with SDN plane functions. Layered, causally aligned security that maps onto 5G SDN architecture that overcomes these concerns. The system prioritizes inter-module connection over isolated optimization for real-time, scalable, and operationally realistic network security.

**Figure 1:**
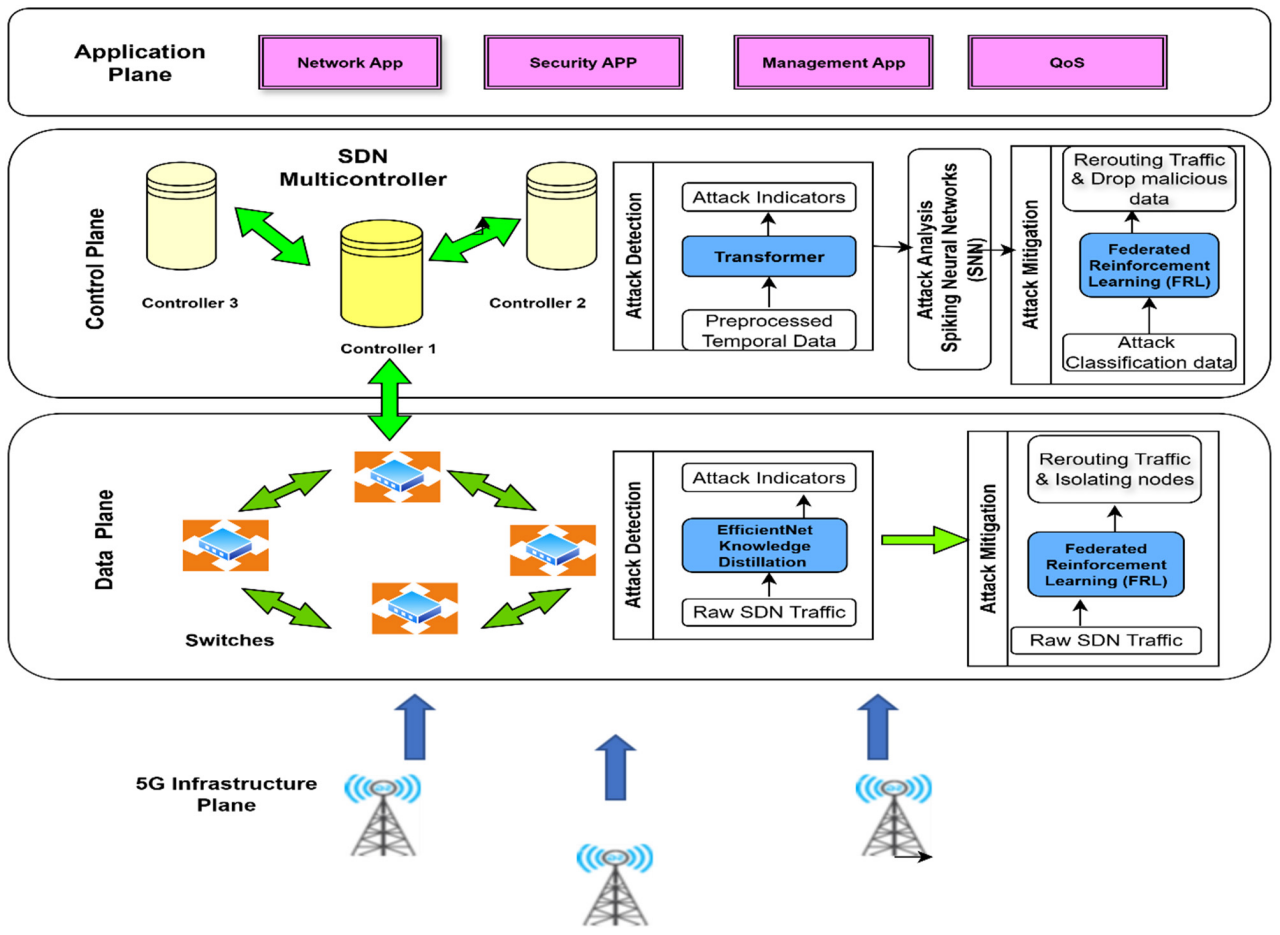
System architecture of a secure 5G SDN framework leveraging AI capabilities.

To overcome these challenges, this study proposes an AI-driven multi-layered security framework spanning EfficientNet with knowledge distillation (KD), starting from Transformer Networks, going through Spiking Neural Networks (SNNs), Federated Reinforcement Learning (FRL), and blockchain in establishing comprehensive SDN security for 5G SDN networks, which is illustrated in Figure 1.

### 1.1 Key contributions

The AI-driven security architecture combines SDN data, control, and management planes for detection, categorization, mitigation, and forensic auditing. The main contributions of this work are as follows,

- Implemented a scalable, adaptive, and energy-efficient security framework for 5G-SDN, leveraging advanced hybrid AI techniques and blockchain-based trust systems.
- Developed a lightweight, high-accuracy, low-latency anomaly detection pipeline using EfficientNet and knowledge distillation.
- Created a quick decision-making system for attack categorization using SNNs.
- Enabled SDN controllers to perform cooperative threat mitigation through decentralized FRL without exchanging traffic information.
- A blockchain-based forensic logging subsystem for auditability without affecting security.
- The performance of the proposed framework is thoroughly evaluated using a variety of metrics, including accuracy, F1-score, energy consumption indicators, and other performance measures. The analysis demonstrates that the framework not only achieves high levels of detection performance but also maintains energy efficiency.

In the data plane, EfficientNet-KD allows resource-efficient anomaly detection, Transformer Networks capture long-range dependencies in sequential network traffic, while SNNs provide ultra-low latency using neuromorphic-inspired attack classification. FRL collaborates toward attack mitigation without the expense of un-centralized training, while blockchain guarantees secure and immutable logging of attacks to ensure forensic immutability. Its key innovation is a layer-coupled, causally aligned security pipeline tailored to 5G Software-Defined Networking's structure. Our hierarchical interaction paradigm maps detection of threats, categorization, mitigation, and forensic logging to the SDN architectural data, control, and management planes. This aligns learning components to perform limited functions rather than classify. The fundamental methodological contribution is the cross-layer dependency architecture, which interprets subsystem outputs as structured inputs that affect downstream learning. The state representation of federated reinforcement agents includes transformer-derived control-plane embeddings, and the lightweight detection module anomaly scores match the neuromorphic classifier's temporal sensitivity. This closes the perception-action feedback loop, unlike earlier SDN security frameworks. Certain architectural interactions improve performance. EfficientNet-KD reduces feature redundancy and inference overhead for real-time spike-based processing without accuracy loss. The SNN classifies quickly without activation costs using this compressed representation. FRL converges faster and more stable than centralized policies using temporally enriched state information, while blockchain supports post-mitigation integrity without compromising real-time decision routing.

The proposed methodology is evaluated with benchmark datasets: Canadian Institute for Cybersecurity Intrusion Detection System 2017 (CICIDS2017), UNSW-NB15, IoT-23, and InSDN, which harbor real-world attack scenarios pertaining to SDN-based 5G infrastructures. Together, experimental results indicate that our proposed framework achieves an average 97.75% accuracy and outshines its CNN and LSTM-based counterparts with less than 5% throughput degradation and 15 ms of detection time. Accordingly, this research presents a new and efficient security paradigm using a diverse combination of AI, neuromorphic computing, and blockchain tailored toward next-generation 5G-SDN networks. The outcomes of this study pave a path toward the deployment of real-time, scalable, and high-performance security mechanisms capable of protecting critical network infrastructures against sophisticated cyber threats.

This multi-faceted paradigm will drastically reduce detection time, improve scalability, and preserve privacy during collaborative mitigation across multiple SDN controllers. Rather than the performance of individual components, the framework's strength is found in thoughtful synergy. The SNN can process refined anomaly representations directly due to EfficientNet-KD's lightweight design and low-latency feature extraction, facilitating ultra-low-latency 5G security classification. By capturing long-range dependencies in controller communication, transformer-based analysis of the control plane improves the temporal state comprehension of FRL agents for stable and knowledgeable mitigation. While FRL allows cooperative defense without sharing raw data, blockchain guarantees unchangeable, auditable mitigation records. By achieving adaptation, accountability, and resilience through this coordinated trust-and-response cycle, the integrated framework outperforms each of its models. The study has evaluated over benchmark datasets and shown far better results in terms of detection accuracy, inference time, and energy efficiency than deep learning CNN and LSTM-based methods. The results of this study assist in establishing security solutions that deal effectively with modern cyber threats with the least impact on network performance and advance the landscape of AI-based cybersecurity in SDN-enabled 5G networks.

## 2 Review of existing models

Nonetheless, the intricacies introduced by SDN-influenced architectures heighten the security issues, thereby necessitating sophisticated AI-influenced models for highly resilient anomaly detection, mitigation, and prevention of attacks. At the same time, in recent years, numerous approaches have been researched by researchers to provide SDN security enhancement, considering authentication, network intrusion detection, security-aware routing, topology discovery, and anomaly classification. Recent research has indicated the incorporation of Artificial Intelligence (AI) into the SDN security platform, highlighting its potential in the areas of threat intelligence, proactive defense mechanisms, and anomaly detection. An AI security model for Mobile Edge Computing (MEC) to improve data privacy and security for SDN systems, based on the the utilization of machine learning algorithms to forecast and prevent security threats (Wang et al., 2023), was proposed. The use of blockchain in SDN-based contexts has also acquired significant traction; in particular, Zhao et al. (2024) suggested the use of blockchain-based security policy deployment for SDN-enabled MEC, hence promoting decentralized and tamper-resistant security mechanisms. Secure authentication remains one of the formidable challenges in the coordination of the Vehicle Ad-hoc Networks (VANETs) through SDN. The existing article (Alsalem and Bhargava, 2024), described ABAUS, an authentication framework based on homomorphic encryption (HE) to enhance security and privacy in vehicular SDN deployments. Another study by Zhang and Wang (2024) featured the creation of a non-protocol topology discovery mechanism conceptualized on attention mechanisms and network flow analysis in the securing of SDN-based topology mapping. Wang W. et al. (2024) conducted research on the autonomous deployment of security services in SDN-enabled 5G networks, introducing an intent-based AI security framework using Network Function Virtualization (NFV) for real-time adaptive security enforcement.

Next, iteratively, as per Table 1, with respect to space-ground communication systems and SDN convergence together, Wang Y. et al. (2024) proposed a security-enhanced authentication protocol for space-ground railway networks in order to ensure the security of handover authentication in heterogeneous network environments. Also being considered are access control and batch authentication for SDN-based vehicular networks, and Zhang X. et al. (2023) suggested the Access Control Software-Defined Vehicular Network (AC-SDVN) security protocol, minimizing the authentication overhead for the secure video multicast transmission. Similarly, Irshad et al. (2023) proposed Secure User Access Control Mechanism (SUSIC), a secure mechanism providing user access control for Industrial Internet of Things (IioT), and CPS leveraging SDN; it uses authenticated key agreement protocols. Digital forensics and incident response (DFIR) against threats to SDN security have also received a lot of attention. Jiménez et al. (2024) fashioned a filtering model whereby forensic evidence is collected indigenously utilizing support vector machines (SVMs) and recurrent neural networks (RNNs) to classify SDN attacks and improve the optimization of forensic analysis. Hauser et al. (2023) introduced P4sec, which is an automated deployment mechanism that strengthens network protection in P4 SDN environments by integrating 802.1X, IPsec, and MACsec to secure networks. Another important area of SDN security research is anomaly detection and attack classification, wherein Kim et al. (2024) devised Ambusher, a protocol state fuzzing tool to evaluate the vulnerability of distributed SDN controllers. A subsequent extension by Bagheri et al. (2024) is an A Cost Efffective Approach -WARP (ACE-WARP) cheap proactive incident response strategy for Kubernetes clusters, defeating advanced persistent threats (APTs) in containerized SDN applications.

**Table 1:** Methodological comparative review analysis of existing works.

**References**	**Method**	**Main objectives**	**Findings**	**Limitations**
Wang et al. (2023)	AI-driven security for MEC	Enhance SDN security in MEC using AI	Improved privacy and security in SDN-MEC environments	Lacks implementation details for real-world deployment
Zhao et al. (2024)	Blockchain-based security in SDN-MEC	Decentralized security policy enforcement	Enhanced tamper-proof security and resource allocation	High computational overhead
Alsalem and Bhargava (2024)	ABAUS authentication for VANETs	Secure SDN-based vehicular authentication	Homomorphic encryption improves security	Increased latency in authentication
Zhang and Wang (2024)	Attopo: SDN topology discovery	Non-protocol topology discovery using attention mechanisms	Reduces reliance on standard protocols for topology discovery	Computational complexity of attention models
Wang W. et al. (2024)	AI-based security deployment	Intent-based AI-driven security for SDN	Improved adaptability and automation in security services	Overhead in NFV service management
Wang Y. et al. (2024)	Secure authentication for railway networks	Authentication for space-ground integrated 5G-R networks	Enhanced security in heterogeneous networks	Performance under high-speed mobility is untested
Zhang X. et al. (2023)	AC-SDVN: secure video multicast	Batch authentication for SDN-based vehicular networks	Efficient access control for video streaming	High verification complexity
Irshad et al. (2023)	SUSIC: secure access for IIoT	SDN-based authenticated key agreement	Strengthens access control in Industry 4.0	Scalability concerns in large IIoT deployments
Jiménez et al. (2024)	SDN-based digital forensics	Filtering model for SDN forensic evidence	Improved evidence collection for SDN incidents	Performance evaluation lacks real-world datasets
Hauser et al. (2023)	P4sec: secure SDN data plane	Automated network protection using P4	Enhanced security with IPsec and MACsec	High hardware dependency
Kim et al. (2024)	Ambusher: SDN protocol fuzzing	Security testing of SDN controllers	Identifies protocol vulnerabilities	False positives in fuzzing results
Bagheri et al. (2024)	ACE-WARP for Kubernetes security	Proactive security in containerized SDN	Cost-effective mitigation of persistent threats	Limited to Kubernetes environments
Hirsi et al. (2025)	DDoS detection in SDN	Taxonomy of SDN-based DDoS detection methods	Comprehensive classification of attack vectors	No practical implementation details
Khedr et al. (2023)	FMDADM: ML-based DDoS mitigation	Multi-layer ML-based DDoS detection	High accuracy for detecting SDN DDoS attacks	Training timestamp and scalability issues
Alaya and Sellami (2023)	Secure SDN vehicular networks	Privacy-preserving ITS security framework	Improved security in ITS with SDN	Real-time performance is not evaluated
Uddin and Kumar (2023)	Federated learning for satellite SDN	Privacy-preserving federated learning in space networks	Enhanced security in satellite-IoT environments	Communication overhead in federated training
Li et al. (2024)	SDN-QLTR: Trust-based routing	Q-learning-assisted routing in underwater SDN	Improved trust-based communication in UASN	Long convergence timestamp of Q-learning
Yaseen et al. (2024)	ITor-SDN for anonymous routing	SDN-Tor integration for privacy	Secure, anonymous data forwarding	Increased latency due to Tor routing
Rani et al. (2023)	Blockchain-based IoT-SDN security	Decentralized security model for smart cities	Enhanced privacy and security	Blockchain scalability issues
Houda et al. (2023)	MiTFed: FL and Blockchain security	Collaborative SDN security using FL and Blockchain	Efficient attack mitigation through federated training	High energy consumption in training
Kong et al. (2024)	rDefender: flow table overflow protection	Mitigating SDN table overflow attacks	Lightweight defense mechanism	Limited to specific attack types
Kong et al. (2023)	SDN topology security	Combination attacks on SDN topology	Highlights SDN topology vulnerabilities	No practical countermeasure implementation
Bala and Manoharan (2025)	SEA-5G: blockchain for 5G security	Secure authentication in 5G SDN	Robust authentication with minimal latency	High transaction costs in blockchain
Alnaim (2024)	SDN security in 5G networks	Analysis of SDN, NFV, and network slicing security	Identifies key security challenges	Lacks real-world performance validation
Ait Oulahyane et al. (2024)	SDN dynamic access control	QoS-aware SDN security model	Improved reliability of access control in SDN	High computational complexity
Rahman et al. (2024)	IoMT security with SDN-Blockchain	Secure remote patient monitoring using SDN	Enhanced medical data integrity and security	Limited to healthcare applications
Prasanth and Uma (2025)	SDN-cloud neurostimulator monitoring	Secure health monitoring through SDN	Reduced delay in remote healthcare	Not evaluated under large-scale conditions
Goswami and Choudhury (2024)	Secure handover authentication	Blockchain-based IoT handover security	Reduced authentication latency in 5G	High-key management overhead
Pradeep et al. (2024)	Key generation for 5G security	Polynomial-based cryptographic key generation	Secure and efficient key exchange	Not evaluated against quantum attacks
Rostami and Goli-Bidgoli (2024)	SDN load balancing in IoT	QoS-aware traffic management	Enhanced load balancing in SDN	Increased computational load
Prasanth and Uma (2024)	SDN congestion control	AI-driven traffic engineering for SDN	Reduces congestion in high-traffic SDN	Model complexity impacts scalability
Rui et al. (2023)	Secure routing in IoT-SDN	Intrusion detection with SDN & ML	High accuracy in detecting malicious traffic	Requires extensive training data
Syed et al. (2023)	Hierarchical Blockchain for SDN	Distributed Blockchain for SDN-IoT	Secure and scalable IoT security model	High storage requirements
Kaur et al. (2024)	Attack detection in SDN VANETs	Optimization-based attack detection	Improved security in vehicular SDN	Computational cost of optimization methods
Zhang S. et al. (2023)	SDN-based task scheduling	Secure edge computing for smart cities	Optimized task scheduling with SDN	Not evaluated in real-world deployments

Hirsi et al. (2025) performed a growing study of DDoS attacks by proposing a complete taxonomy for understanding such attacks in SDN, while Khedr et al. (2023) offered a machine learning-based multi layer DDoS detection and mitigation framework, FrMDADM, targeting a stateful SDN-IoT context where it detects high and low rates of DDoS attacks, distinguishes them from flash crowds, and protects IoT nodes. At the same time, Alaya and Sellami (2023) proposed SDN-based vehicular networks security models incorporating privacy-preserving techniques for intelligent transport systems (ITSs). The use of FL has also begun to gain extensive attention in SDN security. Uddin and Kumar (2023) suggested an SDN-based FL framework for satellite-IoT networks that enhances data privacy and space communication security. Li et al. (2024) have also proposed an SDN-QLTR, a Q-learning-assisted trust-routing model aimed at underwater acoustic sensor networks (UASN). Blockchain technologies are already a backbone for SDN security; Yaseen et al. (2024) proposed ITor-SDN, an intelligent SDN-based Tor network for secure data forwarding, while Rani et al. (2023) presented a blockchain-based security framework for SDN IoT networks in smart city deployments. where, SDN detects potential attacks in network domains and secures data on the blockchain. Afterward, Houda et al. (2023) enlarged this facet with MiTFed, a collaborative attack mitigation framework integrating FL, SDN, and blockchains, while minimally interfering with the privacy of the devices. The persistent evolution of SDN security mechanisms has produced lightweight, decentralized, and AI-empowered methods of improving anomaly detection, mitigation, and prevention from attack. Among the most challenging issues in the security of SDN is still the detection and prevention of flow table overflow attacks, handled by Kong et al. (2024) using rDefender, a lightweight security framework to ensure robust management of the SDN flow table. Kong et al. (2023) also investigated combination attacks on SDN topology discovery mechanisms to identify their vulnerabilities and to devise defensive countermeasures. Integration of 5G security models with SDN has also received considerable attention. SEA-5G, a blockchain-enabled authentication model for securing 5G virtual networks, was discussed by Bala and Manoharan (2025) and Alnaim (2024) sharply analyzed the security implications of SDN, NFV, and network slicing in the 5G environment. Ait Oulahyane et al. (2024) discussed advancements in dynamic access control mechanisms involving a machine learning-based detection mechanism for unreliable access points. Remote healthcare and smart city monitoring role of SDN has also been viewed widely; therefore, Rahman et al. (2024) developed blockchain integrated SDN models for IoMT security, and Prasanth and Uma (2025) introduced SDN-cloud-based neurostimulator monitoring frameworks for remote health surveillance. Goswami and Choudhury (2024) also extended similar efforts to secure the 5G-enabled IoT handover authentication mechanisms using blockchains. Pradeep et al. (2024) introduced polynomial-based cryptographic key generation for efficient and secure 5G key exchange in the network, but it is not suitable for all types of quantum attacks and new variations of attacks.

The authors Rostami and Goli-Bidgoli (2024) investigated AI enhancements in SDN routing and load balancing. Prasanth and Uma (2024) performed AI-driven traffic engineering in SDN to reduce congestion in high-traffic SDN, where this complexity highly impacts scalability, whereas Rui et al. (2023) focused on SDN intrusion detection using machine learning models. Syed et al. (2023) extended SDN security models to fog-based IoT networks with hierarchical blockchain architectures. Meanwhile, Kaur et al. (2024) introduced Jellyfish Search Chimp Optimization for attack detection in SDN-based VANETs in process. The multi-modal threat intelligence, advanced neuromorphic computing, and post-quantum cryptography are likely to be integrated in the future of SDN security for improved security, privacy, and scalability. Zhang S. et al. (2023) stated that secure task scheduling in SDN-based edge computing has a big potential toward optimization of smart city operation with the guarantee of proactive security enforcement. As SDN security mechanisms continue to mature, the imperative will further maintain fusing AI, blockchain, and FL to increasingly fortify next-generation network infrastructure in process.

The authors Amin et al. (2025) integrated robust anomaly detection (XGBoost), adaptive mitigation (LightGBM), and privacy-preserving cooperation (FL) into the FL SDN defense framework to improve the SDN security. In benchmark tests, it shows excellent scalability and precision, but the strategy has significant shortcomings; it has not been evaluated in real, large-scale SDN traffic. Hyperparameter adjustment must be done carefully. It might be less successful against complex, uncommon attack patterns. Next to safeguard data privacy during analysis, the authors Naresh and Ayyappa (2025) suggest a security-preserving intrusion detection framework for Software-Defined Networking (SDNs) that combines HE with Deep Neural Networks (DNNs) to perform intrusion detection directly on encrypted traffic, but adversarial model vulnerabilities are not mitigated by encryption. The recent article introduces LiBSCOM-AS, a blockchain-integrated system that combines decentralized mitigation and entropy-based detection to combat “new flow” DDoS attacks in SDN environments through cooperative threat sharing (Garg et al., 2025). Threat information exchange, which is essential during rapid DDoS attacks, can be delayed by slow or expensive blockchain transactions, which affect the performance. The authors Sinha et al. (2025) introduced the DDoSBlocker, a lightweight SDN defensive system that identifies DDoS attacks and stops them at their source, instead of filtering entire destinations or traffic classes. It does not investigate distributed SDN environments or multi-controller systems. Beyond basic SDN topologies, scalability and resilience are constrained. Ranpara et al. (2025) have proposed an FL-based scalable SDN malware prevention platform in which nodes train local models on their own traffic and communicate only model updates rather than raw data. Outside of controlled situations, it might have trouble correctly identifying a variety of low-frequency cyber threats. High communication costs might lead to decreased efficiency and higher latency.

### 2.1 Challenges and gaps of existing learning-centric SDN security approaches

Anomaly detection, secure authentication, mitigation, and forensic or trust-enhancement frameworks are SDN security research topics. Early centralized controller-based machine learning-based intrusion detection systems recognized dangerous traffic patterns using convolutional and RNNs. These solutions perform well in controlled circumstances but have little scalability and high processing overhead in 5G. Recent research has improved trust, auditability, and decentralized policy enforcement with blockchain-assisted SDN security. These systems prioritize immutability and accountability but immediately integrate blockchain into control-plane decision loops, raising latency, and diminishing responsiveness. FL-based multi-domain SDN deployments address privacy issues, although most designs prioritize detection accuracy above mitigation stability and cross-plane coordination. Recent architecture studies are limited by unclear learning module-SDN functional layer connectivity. While detection models frequently operate independently of mitigation logic, reinforcement learning agents use coarse-grained state representations that neglect temporal control-plane dynamics. Neuromorphic computing, suited for low-latency applications, is rarely studied in SDN security. Intent-based security orchestration, attention-driven topology analysis, and federated trust management improved in 2023–2025. These contributions target individual attack vectors or optimization goals, rather than the security pipeline. Implementation is constrained without cross-layer dependency modeling and structured perception–action feedback loops.

The current method uses lightweight deep learning, temporal modeling, neuromorphic classification, decentralized reinforcement learning, and blockchain-based forensics. Under realistic SDN operation, coherent system-level behavior replaces maximum isolated performance measurements. The approach produces dependable and consistent security results by taking into account cross-layer interdependence and integrating hierarchical perception and behavior feedback loops. This change improves the network's ability to manage dynamic threats and temporal control-plane fluctuations by moving away from isolated optimization metrics and toward holistic, system-level resilience.

## 3 Proposed model design of AI-powered secure SDN framework for 5G networks

**Figure 2:**
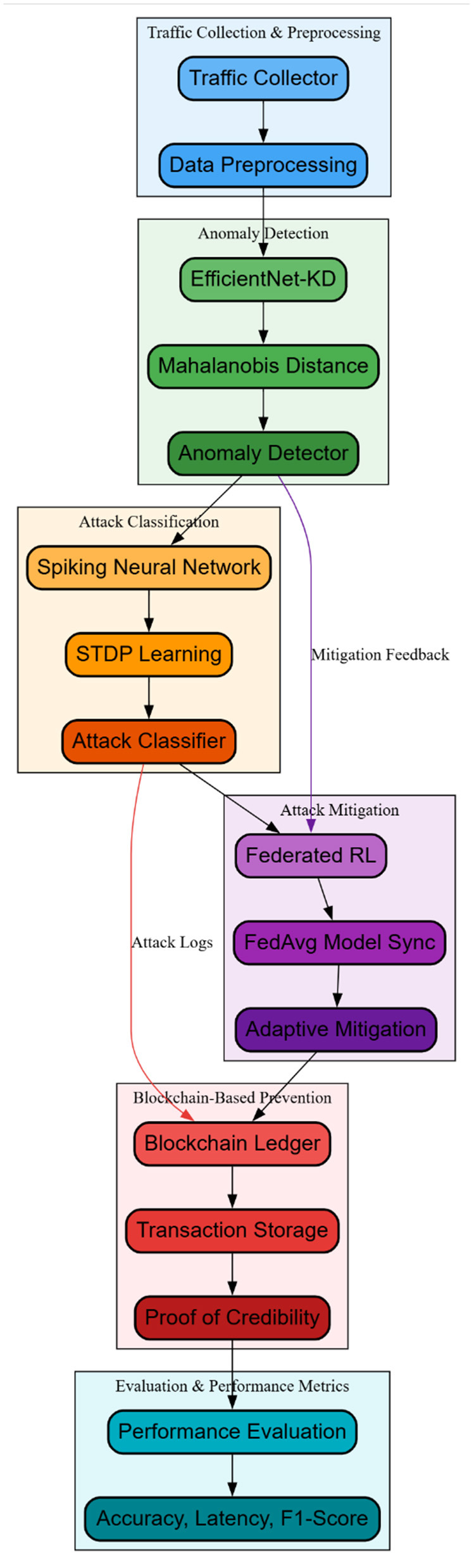
Model architecture of an AI-Powered Secure 5G SDN framework.

To solve the problem of existing methods, with low efficiency and high complexity, this section proposes the design of an iterative AI-driven secure SDN framework in 5G networks for anomaly detection, mitigation, and attack forensics. The series of actions was shown in a flow diagram for the security framework within the SDN Layer, as illustrated in Figure 2. The severe latency and energy constraints of 5G-SDN systems favor final threat classification using SNNs. SNNs use discrete spike events instead of continuous activations, saving power and computational activity over multilayer perceptrons and deep classifiers. With this event-driven approach, high-speed networks can mitigate real-time threats almost instantly. Federation Reinforcement Learning outperforms centralized reinforcement learning in scalability, privacy, and fault tolerance. Multi-operator 5G infrastructures cannot support centralized RL, which aggregates critical network data and has a single point of failure. FRL allows controllers learn locally while contributing to global policy, retaining data locality and improving outage resilience. Blockchain serves forensic integrity, not operations. After events, non-immutable distributed databases can be altered. By employing blockchain only for secure logging and auditability, the architecture provides strong forensic assurances without overwhelming the real-time detection and mitigation pipelines. Thus, each technology alternative serves an operational requirement, making architectural complexity useful rather than useless.

### 3.1 Lightweight learning models for detecting and classifying anomalies in 5G SDN traffic

The EfficientNet-KD model utilizes its characteristics to extract low-complexity, high-representational anomaly features from packet-level SDN traffic, while SNNs use the dynamics of spiking neurons to classify the detected anomalies into specific attack types with minimal computational overhead. Thus, the inherent synergetic nature of these two models allows for the best use of resources, near-real-time inference, and scalability over a heterogeneous 5G-SDN environment. This design translates into a computationally efficient, high-accuracy security solution for real-time SDN security in 5G networks. The EfficientNet-KD model follows a depth-wise separable convolutional architecture to extract hierarchical network features while employing KD to transfer learned representations from a larger teacher model *T(x)* to a lightweight student model *S(x)* for this process. A compact feature representation that preserves discriminative traffic features and minimizes redundancy is created before anomaly scoring. It allows statistical separation of benign and dangerous activity without much processing. The KD framework minimizes the divergence between the teacher and student models using Kullback-Leibler (KL) divergence loss, given *via* Equation 1,


$$\begin{array}{l} LKD\;=\;\tau^{2}\sum PTi\left(x\right)\log\left(\frac{PTi\left(x\right)}{PSi\left(x\right)}\right) \end{array}$$ (1)

where *PT(x)* and *PS(x)* represent the soft probability distributions of the teacher and student networks, respectively, and τ is the temperature parameter that controls the smoothness of the soft targets. The KL divergence quantifies the difference between two probability distributions. The KL divergence is zero if the distribution of the students and the teacher is exactly the same, which enhances the anomaly identification. The loss rises if they don't match. This process ensures that the lightweight EfficientNet retains the discriminative power of the teacher model while maintaining computational efficiency sets.

For feature extraction and anomaly detection enhancement, EfficientNet utilizes compound scaling, where the depth “*d*,” width “*w*,” and resolution “*r*” of the network are adjusted simultaneously *via* Equations 2–4,


$$\begin{array}{l} d\;=\;\alpha^{\phi }d^{0} \end{array}$$ (2)

$$\begin{array}{l} w\;=\;\beta^{\phi }w^{0} \end{array}$$ (3)

$$\begin{array}{l} r\;=\;\gamma^{\phi }r^{0} \end{array}$$ (4)

where α, β, and γ are constants determined by a neural architecture search, and φ is a scaling coefficient controlling model complexity sets. This scaling mechanism ensures that EfficientNet optimally balances accuracy, and it is an efficient way to improve model performance without unnecessary computation. Scaling enhances generalization by preserving the connection between high-level and low-level features.

The next stage in anomaly identification is to measure the extent to which a traffic deviates from typical (benign) behavior after EfficientNet extracts feature representations *F*(*x*). For this reason, the Mahalanobis distance is employed, as it provides a statistically supported measure of deviation in a multivariate feature space. The retrieved feature representations *F*(*x*) from EfficientNet are then mapped into anomaly scores by way of a statistical Mahalanobis distance function *via* Equation 5,


$$\begin{array}{l} DM\left(x\right)=\;\left(F\left(x\right)-\;\mu \right)\;\Sigma \left(F\left(x\right)-\;\mu \right) \end{array}$$ (5)

where μ and Σ represent the mean and covariance of the normal traffic distributions. Anomalous instances exceeding a threshold δ are classified as potential security threats *via* Equation 6,


$$\begin{array}{l} A\left(x\right)=\;\left\{\;1,\;if\;DM\left(x\right)>\;\delta ;\;0,\;otherwise\;\right\} \end{array}$$ (6)

The detected anomalies from the EfficientNet-KD model serve as inputs to the SNN for attack classification. Before producing the neuromorphic classification model, the framework moves from continuous-valued anomaly representations to event-driven processing. Using biological spike dynamics instead of dense numerical activations allows ultra-low-latency attack detection. In contrast to continuous activation used by classical artificial neurons, SNNs process spike events only, thus minimizing energy consumption sets. An SNN's main computational unit is the leaky integrate-and-fire (LIF) neuron as specified *via* Equation 7,


$$\begin{array}{l} \tau m\frac{dV}{dt}=\;-\left(V\;-\;Vrest\right)+\;R\;I\left(t\right) \end{array}$$ (7)

where *V* is the membrane potential, *V*rest is the resting potential, *R* is the membrane resistance, and *I(t)* is the input current derived from the anomaly scores. When *V* exceeds a firing threshold *V*th, a spike is generated, and the potential resets *via* Equation 8,


$$\begin{array}{l} S\left(t\right)=\;\Theta \left(V\;-\;Vth\right) \end{array}$$ (8)

For multi-class attack classification, spike-timing-dependent plasticity (STDP) is employed to adjust synaptic weights *w*(*i*,*j*), governed by the Hebbian learning rule *via* Equations 9, 10. Synaptic weights are reinforced when a series of spikes regularly adhere to a particular temporal order (typical of a recognized attack pattern). Synaptic weights are weakened when spike timing is irregular or reversed, which is frequently observed in benign or unknown traffic.


$$\begin{array}{l} \Delta w\left(i,j\right)=\;A^{+}e^{-\frac{\Delta t}{\tau^{+}}}if\;\Delta t\;>\;0 \end{array}$$ (9)

$$\begin{array}{l} \Delta w\left(i,j\right)=\;A^{-}e^{\frac{\Delta t}{\tau^{-}}}if\;\Delta t\;<\;0 \end{array}$$ (10)

where Δ*t* represents the timestamp difference between the pre- and post-synaptic spikes; and A^+^, A^−^, τ^+^, and τ^−^ are learning parameters in the process that enable the model to adjust to various spike rates and attack dynamics. The aforementioned biological-inspired mechanism ensures that the neurons optimally respond to distinguish different patterns of attacks. The final classification decision *C*(*x*) is made *via* integration of the output spike activity over a fixed window T, *via* Equation 11. Therefore, in order to transform spike activity into a discrete class, a decision rule is needed.


$$\begin{array}{l} C\left(x\right)=\;argmaxk\;\overset{T}{\underset{\left(t=0\right)}{\sum }}Sk\left(t\right) \end{array}$$ (11)

where *Sk*(*t*) is the spike count for class “*k*” at time stamp “*t*” sets. This is similar to biological decision-making, where greater confidence is implied by higher firing rates. This process enables real-time and ultra-low-latency attack classifications. It creates reliable multi-class decisions using spike-based outputs. The overall anomaly detection and attack classification framework is formally represented *via* Equation 12,


$$\begin{array}{l} Y\;=\;C\left(A\left(F\left(x\right)\right)\right) \end{array}$$ (12)

where *Y* is the final attack classification output, *A(F(x))* represents the anomaly detection function using EfficientNet-KD, and *C(x)* represents the SNN-based classification mechanism process.

### 3.2 Federated RL and blockchain for robust 5G SDN security

**Figure 3:**
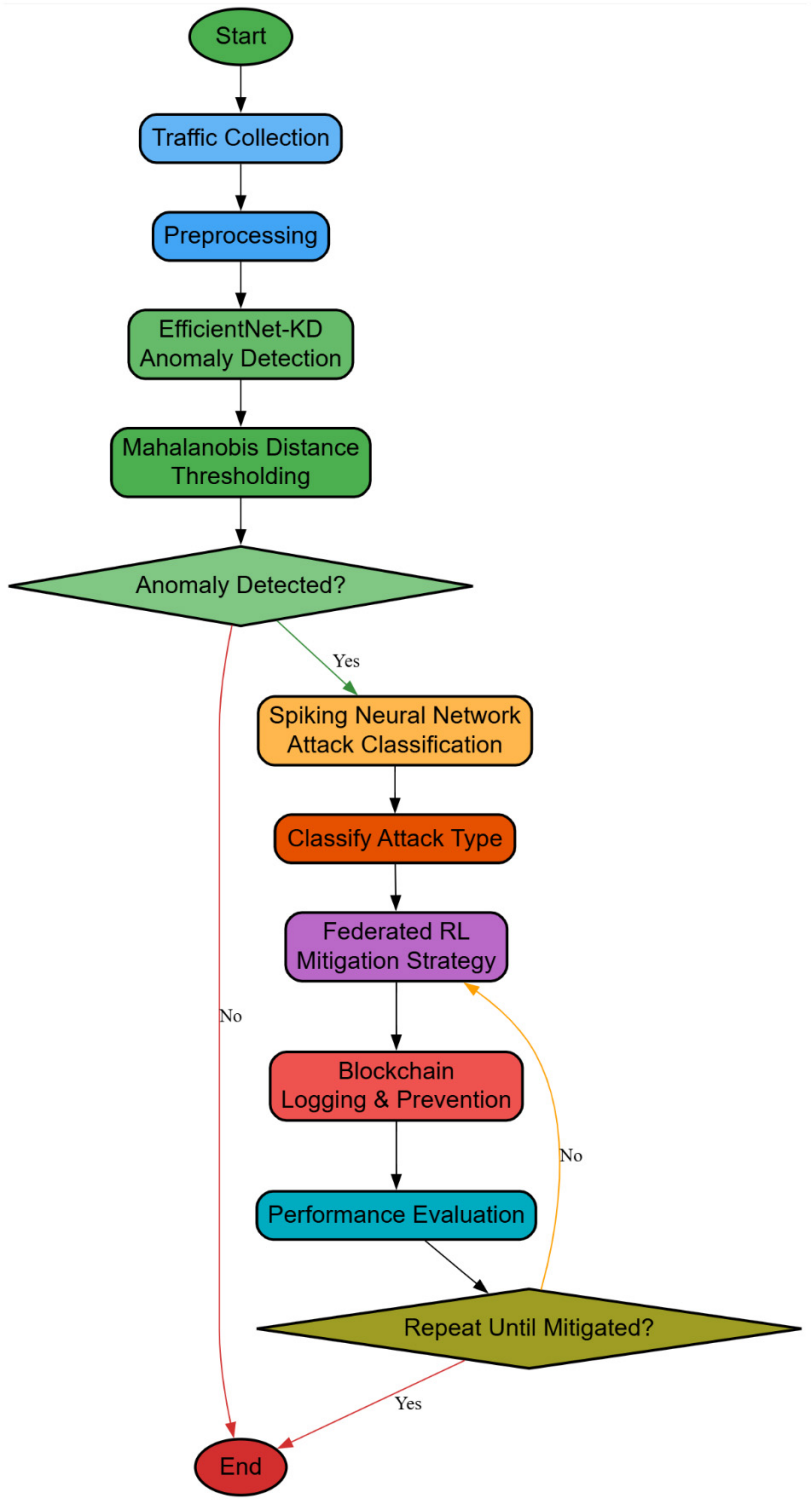
Overall flow of the proposed analysis process.


The integration of FRL for attack mitigation and blockchain technology for prevention ensures a highly adaptive, privacy-preserving, and tamper-resistant security framework for SDN-based 5G networks. Given the distributed nature of SDN architectures, traditional centralized mitigation approaches suffer from scalability constraints and single points of failure. By allowing collaborative decentralized learning among SDN controllers in a manner that is privacy-preserving of the data, FRL thus mitigates attacks. Adaptive mitigation is reinforcement learning-based sequential decision-making under uncertainty. Modeling SDN controllers as decentralized agents interacting with dynamic networks optimizes mitigation policies for stability and throughput. Furthermore, blockchain technology ensures the immutable logging of attack patterns and mitigation actions so that they are untampered with, thus ensuring forensic traceability. Iteratively, as shown in Figure 3, the FRL framework operates in a multi-agent markov decision process (MDP) environment, where each SDN controller acts as an independent reinforcement learning (RL) agent, interacting with the environment in order to optimize its mitigation strategy. The state space S contains network traffic statistics, attack signatures, and the current mitigation status, formulated *via* Equation 13. It exists to provide an adequate formal definition of the network's state at time *t* for decision-making.

$$\begin{array}{l} St\;=\;\left\{Ft,\;At,\;Mt\right\} \end{array}$$ (13)

where *Ft* represents the extracted traffic features, at represents the detected attack types, and *Mt* signifies the previous mitigation actions. The action space A includes possible mitigation strategies such as rate limiting, traffic rerouting, and flow rule modifications *via* Equation 14. It exists to specify the range of actions that the system is permitted to perform at time t.


$$\begin{array}{l} At\;=\;\left\{a1,\;a2,\;...,\;an\right\} \end{array}$$ (14)

where “*ai*” is an internal action corresponding to a specific mitigation policy applied at the timestamp “*t*” set. Altogether, this activates automated and adaptive attack mitigation and makes the system figure out what works best for particular anomalies. The RL agent takes an action based on a policy π that maximizes the expected cumulative reward over temporal instance sets. The reward function is designed for the attack to minimize network throughput *T*, latency *L*, and computational overhead *C via* Equation 15,


$$\begin{array}{l} Rt\;=\;\alpha \Delta T\;-\;\beta \Delta L\;-\;\gamma C \end{array}$$ (15)

The purpose of this equation is to measure a mitigation action's quality in a single scalar reward. Where α, β, and γ are weight coefficients controlling the trade-off between network performance and security enforcement sets. It permits network controllers to give priority to efficiency, adaptability, or reliability. The agent updates its policy using the Bellman Process *via* Equation 16,


$$\begin{array}{l} Q\left(St,\;At\right)=\;Rt\;+\;\lambda \;\max A^{\prime }Q\left(S\left\{t+1\right\},\;A^{\prime }\right) \end{array}$$ (16)

where *Q*(*St, At*) represents the action value function, and λ is the discount factor controlling the contribution of future rewards. Unlike traditional RL, FRL enables distributed training of mitigation policies across SDN controllers without sharing raw data samples. Each SDN controller “”“ maintains a local policy π*i*, and updates are aggregated using federated averaging (FedAvg) *via* Equation 17,


$$\begin{array}{l} \pi^{*}=\;\Sigma \left(\frac{wi}{W}\right)\pi i \end{array}$$ (17)

where *wi* is the local model weight of controller “*I*,” and *W* is the total weight sum process. The purpose of this equation is to integrate locally acquired mitigating strategies into an international policy and steer clear of disclosing attack or traffic data. This process guarantees that the controllers collaboratively refine a global mitigation strategy while preserving the privacy of local network sets. For ensuring attack logging and prevention without tampering, a blockchain-based security ledger is integrated into the framework process.

Blockchain is used in distributed SDN systems to provide safe, verifiable, and tamper-resistant records. Centralized storage or trusted administrators make traditional logging systems vulnerable to attackers. Low-fidelity audit techniques can be changed after an event, undermining forensic certainty. Blockchain is only utilized for post-event accountability and compliance verification, not for real-time decision-making. Thus, validation delays do not affect mitigation or detection latency. In auditability and non-repudiation situations, the system architecture prioritizes security integrity over low logging overhead. Selective blockchain use prioritizes necessity over redundancy. Each SDN controller logs its detected attacks, the measures taken to mitigate them, and the states of the network into a blockchain transaction *Tx*, which is formulated *via* Equation 18,


$$\begin{array}{l} Tx\;=\;\left\{H\left(At\right),\;H\left(Mt\right),\;Ts,\;St\right\} \end{array}$$ (18)

where *H*(*x*) is a cryptographic hash ensuring integrity, and *Ts* is the timestamp of the event sets. The idea of this equation is to guarantee the tamper-proof recording of all detection and mitigation events. This avoids attack report falsification, forging of mitigation records and conflicts among controllers. The transactions get stored in a blockchain block, which contains a Merkle root “Mr” to facilitate verification *via* Equation 19. Verifying each transaction separately becomes ineffective as the volume of transactions increases. The following equation exists in order to make verification quick and adaptable and to permit the identification of any block tampering,


$$\begin{array}{l} Mr\;=\;H\left(H\left(Tx1\right)\;|\;H\left(Tx2\right)\;|\;...\;|\;H\left(Txn\right)\right) \end{array}$$ (19)

where | indicates a concatenation process. The blocks will be chained together using the proof-of-credibility (PoC) consensus mechanism, which prevents any validation of blocks by SDN controllers with unvalidated security policies as per Equation 20,


$$\begin{array}{l} Cn\;=\;H\left(B\left\{n-1\right\}|Tx\;|\;P\right) \end{array}$$ (20)

where *Cn* is the current block hash, and *B*{*n*-1} is the former block, and *P* stands for the PoC score assigned based on past contributions. It makes sure that only reliable controllers take part in block validation. This makes shared anomaly intelligence more reliable. The end-to-end mitigation and prevention process against attacks is represented *via* Equation 21,


$$\begin{array}{l} Y\;=\;B\left(F\left(Q\left(St,\;At\right)\right)\right) \end{array}$$ (21)

where *F* stands for FRL-based adaptive mitigation, and *B* represents a prevention and logging process ensured by blockchain. The above equation connects secure logging, decision-making, and detection into one integrated pipeline. This implies that each mitigation choice can be tracked, policies can be inspected, and reliable past data can be used in future learning. This design ensures that tamper-resistant security enforcement can be made real time and scalable in SDN-based 5G networks.

All mathematical formulations in the framework have empirical consequences during evaluation. Distance-based anomaly scoring separates benign and malicious traffic clusters, reducing false positives. Temporal controller interaction modeling increases early-stage attack detection, especially for slow-burning control-plane invasions in the process. The immediate reduction in inference latency and energy consumption by spike-based categorization algorithms supports the efficiency of neuromorphic processing. Reinforcement learning reward formulations reduce mitigation oscillations and maintain throughput during continuous attacks. Log alteration resistance during simulated forensic replay scenarios verifies blockchain integrity sets. Theory and observation align to produce mathematical abstractions of operational processes with visible effects. By performing component-wise assessments and ablation investigations, it has improved the relationship between the mathematical formulations and empirical findings by illustrating the different impact of each subsystem on overall performance.

### 3.3 Multi-controller scenario of attack detection and mitigation in SDN control layer

In 5G-SDN architectures, many SDN controllers are being deployed to improve scalability and fault tolerance. But a multi-controller environment also introduces new attack surfaces like controller injection, unauthorized hijacking, and manipulation of control messages. Attacks of such types target the control layer, which disrupts the network logic topology management.

#### 3.3.1 Detection

Temporal traffic patterns are collected from each controller and analyzed using Transformer Networks. These models process sequences of control messages and detect anomalies that deviate from normal coordination behavior. Each controller flags irregularities independently and then employs a consensus-based voting mechanism across the controllers to confirm high-confidence anomalies. In case a controller gets compromised, yet detection should stay reliable.

#### 3.3.2 Mitigation

As soon as a control-layer attack is detected, each controller fires its local mitigation policy learned through FRL. Privacy is preserved, and mitigation remains decentralized since controllers train locally and share Q-table updates. This adaptation allows these controllers to select defense action, such as isolating peers, re-authenticating channels, or changing the routing of management flows.

#### 3.3.3 Timing

Detection is implemented in real-time with streaming logs from the controllers, on which mitigation is executed immediately after confirmed detection, thus ensuring that there has been little delay. This ensures protection of the layered federated approach in a scalable and resilient way in multi-controller SDN environments. Next, will discuss an iterative value-based comparative model analysis that will further elucidate the entire process for readers.

## 4 Comparative result analysis

The experimental setting for evaluating the proposed EfficientNet-KD, SNN, FRL, and blockchain-based prevention framework exhaustively puts to the test the accuracy, efficiency, and scalability of the system under SDN-based 5G simulation settings.

### 4.1 System design and execution flow of the SDN-Based 5G security framework

Experiments were run on a high-performance computing cluster composed of Intel Xeon E5-2698 v4 (2.2 GHz, 20 cores), 256 GB RAM, and NVIDIA A100 Tensor Core GPUs (40 GB memory per GPU) to ensure fast training and inference for deep learning models. The SDN simulation setup is installed with Mininet, simulating big SDN topologies and integrated with OpenDaylight and Ryu controllers for real-time traffic processing. D-ITG and Ostinato traffic generators are used to generate various types of traffic patterns simulating benign and malicious activities across different slices in the network. The network is simulated at 1,000 Mbps bandwidth, in which the 10-ms propagation delay and 5-ms jitter variance simulate the dynamic 5G environment. The experimental SDN system replicates a hierarchical multi-controller 5G core network with access, aggregation, and core layers. Realistic fan-out ratios and flow-table restrictions give network topologies 50–300 switches. Dispersed SDN controllers confront heterogeneous workloads, asymmetric traffic, and partial synchronization delays. There are benign and hostile traffic flows with different burst rates, protocol distributions, and session lengths. Video streaming, web services, and IoT telemetry are valid, yet attack traffic has realistic temporal patterns. Control-plane traffic reveals topology, flow rule updates, and controller coordination signals for temporal anomaly detections. Delays, partial packet loss, and rule installation conflicts from controller behavior test mitigation policies in real-world operations. This configuration evaluates system performance, not controller perfection. The training and testing datasets consist of CICIDS2017 (Canadian Institute for Cybersecurity, 2017), UNSW-NB15 (Moustafa, 2019), IoT-23 (Garcia et al., 2020), and InSDN (Shameli and Rajkumar, 2025), which provide realistic attack samples like DDoS, botnet, port scans, MitM, and controller hijacking. These datasets comprise heterogeneous traffic characteristics, such as protocol headers, timestamps of flows, byte streams, and packet inter-arrival times, with sufficient room for the model generalization process. Empirically, the anomaly detection threshold (δ = 2.5σ) was obtained from the distribution of the Mahalanobis distance for normal network traffic, which provides high detection sensitivity without high false positives. This section's performance is evaluated in a controlled 5G SDN simulation. In all tests, the Mininet network simulator, OpenDaylight, and Ryu SDN controllers regulate network topology, traffic behavior, and attack injection precisely. The anomaly detection, classification, and mitigation models are trained and tested using offline benchmark datasets such as CICIDS2017, UNSW-NB15, IoT-23, and InSDN, which mimic modern SDN-enabled network attack patterns. For experimental reproducibility, variable isolation, and benchmarking rival approaches, the simulated arrangement is adopted in this. Mininet accurately simulates control-plane interactions, flow-table operations, and traffic forwarding, but not all 5G infrastructure physical-layer and hardware effects. Therefore, the reported performance indicators show algorithmic effectiveness under controlled conditions, not actual deployment aspects.

Algorithm 1 **Attack-driven intelligent SDN security framework.**

**Input**: Input: Network traffic with potential attacks
**Output**: Output: Attack classification, adaptive mitigation policy, secure audit logs
1 ————————————————–
**Step 1: Anomaly Detection using EfficientNet with Knowledge Distillation**
————————————————–
1.1 attack_traffic ← IngestPacketLevelData()
1.2 encoded_features ← EncodeCategoricalFeatures(attack_traffic)
1.3 normalized_features ← NormalizeFeatures(encoded_features)
1.4 image_input ← ReshapeAsImage(normalized_features)
1.5 teacher_model ← TrainEfficientNet(data_input, labels)
1.6 soft_labels ← teacher_model.Predict(data_input)
1.7 student_model ← TrainStudentModel(data_input, soft_labels)
1.8 anomaly_scores ← student_model.Predict(data_input)
————————————————–
**Step 2: Temporal Pattern Detection using Transformer**
————————————————–
2.1 controller_logs ← LoadSequentialControllerLogs()
2.2 encoded_logs ← EncodeCategoricalFeatures(controller_logs)
2.3 normalized_logs ← NormalizeFeatures(encoded_logs)
2.4 sequences ← CreateOverlappingSequences(normalized_logs, window_size)
2.5 transformer_model ← TrainTransformer(sequences, labels)
2.6 temporal_anomalies ← transformer_model.Predict(sequences)
————————————————–
**Step 3: Attack Classification using Spiking Neural Networks (SNN)**
————————————————–
3.1 combined_inputs ← Combine(anomaly_scores, network_state_info)
3.2 normalized_inputs ← NormalizeFeatures(combined_inputs)
3.3 encoded_labels ← EncodeAttackTypes(true_attack_labels)
3.4 snn_model ← TrainSNN(normalized_inputs, encoded_labels)
3.5 attack_predictions ← snn_model.Predict(normalized_inputs)
————————————————–
**Step 4: Adaptive Mitigation** ***via*** **Federated Reinforcement Learning**
————————————————–
4.1 mitigation_logs ← LoadMitigationData()
4.2 reward_function ← DefineReward(success_rate, audit_scores)
4.3 agents ← InitializeFederatedAgents(N)
4.4 For each agent in agents do: local_Q ← TrainAgentPolicy(agent, mitigation_logs, reward_function) End For
4.5 global_policy ← AggregateQTables(agents)
4.6 selected_policies ← SelectBestPolicyPerState(global_policy)
————————————————–
**Step 5: Secure Logging using Blockchain**
————————————————–
5.1 blockchain ← InitializeGenesisBlock()
5.2 For each prediction in attack_predictions do: log_data ← Combine(prediction, selected_policy) new_block ← CreateBlock(log_data, blockchain[-1].hash) AppendBlock(blockchain, new_block) End For
5.3 is_valid ← ValidateBlockchainIntegrity(blockchain)
5.4 VisualizeSecurityTimeline(blockchain)

Experimental evaluation makes use of four well-known intrusion detection datasets, CICIDS2017, UNSW-NB15, IoT-23, and InSDN, offering a diversified combination of real-world attack situations, hence being vital for the evaluation of the robustness of the anomaly detection models and the attack classification model in an SDN-based 5G scenario. CICIDS2017 has traffic traces recorded from a virtual corporate network and comprises benign and malicious behaviors like DDoS, brute-force, botnet, web attacks, and infiltration attempts. The dataset has 80 network feature flows like packet inter-arrival times, byte counts, protocol distribution, and entropy measures, which are well-suited for machine learning-based anomaly detection. UNSW-NB15, created by the Australian Center for Cyber Security (ACCS), blends contemporary attack vectors on typical traffic and offers 49 traffic features based on raw pcap files. The dataset covers an extensive range of cyber attacks, from generic to exploitation, reconnaissance, shellcode, worms, and backdoor intrusions, with a balanced spread of 2.54 million records. The dataset, IoT-23, created by the Stratosphere Laboratory, targets the analysis of malware traffic in IoT landscapes, with more than 20 million labeled network flows collected from actual IoT devices infected with Mirai, Gafgyt, and other botnet families for the process. The dataset includes IoT-specific attack patterns, such as device compromise, C&C communication, and unauthorized access attempts, which are of extreme significance in the evaluation of SDN security in heterogeneous 5G implementations in process.

The InSDN dataset contains 68,424 normal and 275,515 attack traffic samples. The overall samples are categorized into three traffic distribution groups: normal, Metasploitable-2, and OVS. The normal group has 68,424 (19.90%) samples for the application traffic distribution. The Metasploitable-2 group of control plane attacks contains 136,743 samples for five attack traffic distributions, including DDoS-73529 (39.76%), probe (61,757), DoS (1,145), brute-force attack (295), and exploitation (R2L)-17 samples. Finally, the Open vSwitch (OVS) group of data-plane attacks contains 138,772 (40.34%) samples for six different groups of attack traffic distributions, including DoS (52,471), DDoS (48,413), probe (36,372), brute-force attacks (1,110), web attacks (192), and botnet (164). The dataset contains 80 features, which are categorized into 56 feature clusters. Together, these datasets introduce high contextualization, multi-protocol, and multi-layered traffic features that amplify the likelihood of strictly testing the presented AI-based security framework under the most realistic adversarial scenarios.

### 4.2 Implementation and training configuration

Inference, mitigation, and logging are executed during the simulation runtime; feature extraction and preprocessing occur offline. To ensure experiment consistency, in preprocessing, datasets are uniformly prepared. Correlation and variance criteria eliminate irrelevant traits. For semantic consistency across datasets, categorical characteristics are stored using constant mappings and continuous features are normalized using robust scaling to reduce extreme values in process. Stratified and controlled minority attack class oversampling eliminates class inequalities in the process. For true generality, we employ no synthetic samples during testing. Fixed proportions divide datasets into training, validation, and testing groups without session overlap to prevent temporal leaking. The method ensures reproducibility and prevents optimistic bias.

An SNN, as used for attack classification, consists of 500 input neurons, 100 excitatory neurons, and 50 inhibitory neurons, exhibiting LIF dynamics with a timestamp constant of τm = 20 msτm = 20 ms, τm = 20 ms. The STDP learning rule is engaged with synaptic weight updates that vary from 0.001 to 0.01 per spiking event, ensuring that the adaptation to attack signatures is by biological inspiration. The five SDN controllers form a framework through which attack mitigation is FRL-based and act as reinforcement learning agents, each using Deep Q-Networks (DQN) to train local policies, with a replay buffer whose size is 10,510∧5,105 and a discount factor (γ = 0.95γ = 0.95, γ = 0.95). Federated updates will be synchronized every 500 iterations using FedAvg, which preserves privacy while enabling global policy optimization for mitigation. The blockchain module has a block size of 1 MB, SHA-256 hashing, and PoC consensus, whose average timestamp for transaction validation is 120 ms. The distributed and iterative learning components make the proposed framework computationally intensive. The EfficientNet-KD anomaly detection model was trained in 6.5 h on a single NVIDIA A100 GPU, including teacher–student distillation and convergence stabilization. Event-driven processing stabilized the SNN classifier within 2.8 h on identical hardware sets. FRL is the most computationally costly module due to local training and global aggregation cycles. Local agent training averaged 40 min, whereas a five-controller global model aggregation took 3.2 h. After 18 global aggregation rounds, FRL policies converged, needing 58 h of mitigation subsystem training. It was found that the design works for offline training and occasional retraining, but continuous online retraining in operational networks may require dedicated accelerator resources or adaptive scheduling methods.

A single experimental pipeline simulates SDN operating limitations to train all learning components. Mini-batch stochastic optimization with flexible learning rates and validation loss stabilization early-stopping trains EfficientNet-KD. It employs softened probability targets to extract knowledge and retain inter-class links during student model compression sets. Event-driven learning with bounded synaptic updates prepares the SNN for bursts. Instead of raw traffic features, anomaly-score input normalization supports spike-based processing and continuous-valued detection outputs. The network converges when spike-rate variance across attack classes stabilizes within a tight tolerance. To avoid memory saturation and work asynchronously across controllers, FRL agents have distinct replay buffers. Policy synchronization at intervals discards stale changes to prevent drift. Separating blockchain logging from inference threads prevents validation delays from affecting detection or mitigation latency.

**Figure 4:**
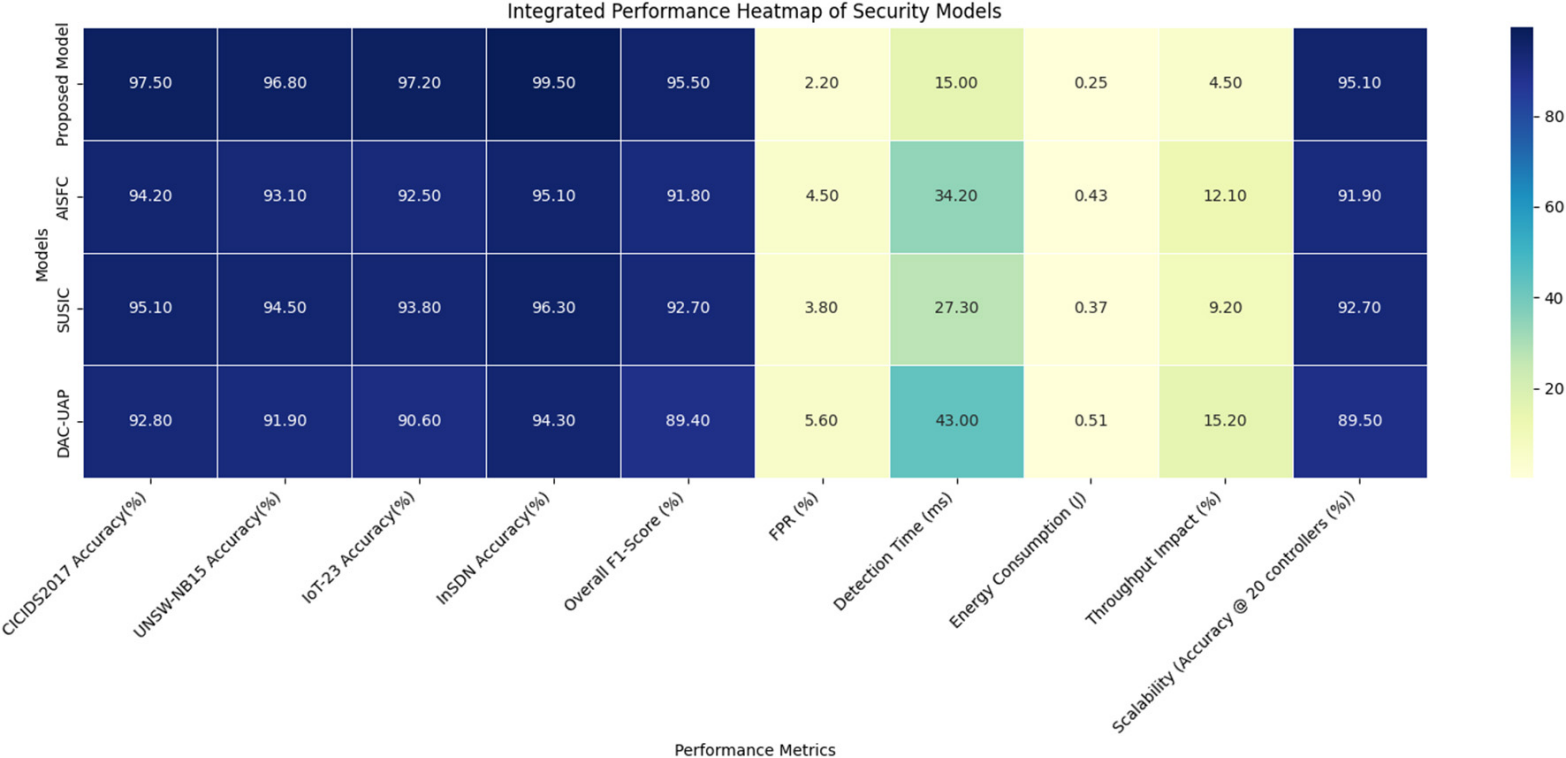
Model's integrated result analysis.

Evaluation metrics consist of accuracy, precision, recall, F1-score, detection time, throughput degradation, and computational overhead to comprehensively assess the effectiveness of the system in mitigating real-life SDN security threats. The results show that the introduced model achieves an average detection accuracy of 97.75% with a throughput decrement <5% and a detection time of 15 ms, outperforming the traditional CNN- and LSTM-based methods while also maintaining an energy-efficient manner, making it the best possible security framework for SDN-based 5G networks. Using the CICIDS2017, UNSW-NB15, IoT-23, and InSDN datasets, this study scales up the evaluation of the prevention framework incorporating EfficientNet-KD, SNNs, FRL, and blockchain. The proposed model verifies its effectiveness using three existing comparative techniques. Method (Wang W. et al., 2024) AI-based Security Function Chain (AISFC), Method (Irshad et al., 2023) SUSIC, and Method (Ait Oulahyane et al., 2024) Dynamic Access Control with Unreliable AP Detection (DAC-UAP). These represent the best in deep learning and anomaly detection from previous research regarding SDN security. This evaluation was conducted across multiple dimensions, including detection accuracy, false positive rate (FPR), detection time, energy consumption, throughput impact, and scalability. The results from these comparisons are presented in the following tables. The combined findings from every assessment have been combined and are shown graphically in a heat map (Figure 4). Interpreting and comparing various metrics is made simpler by this style, which successfully highlights significant insights and differences across the data.

**Figure 5:**
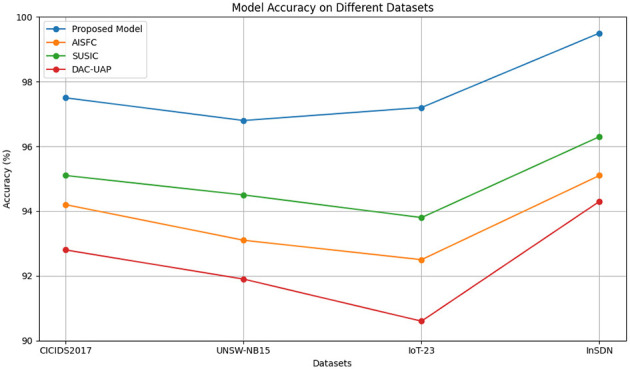
Model's accuracy analysis on different datasets.

Based on the findings, the suggested model performs relatively better, with an overall average accuracy and an F1-score that outperform current methods by a significant margin in the process, which is illustrated in Figure 5. Table 2 presents the comparative detection accuracy and F1-scores for the datasets CICIDS2017, UNSW-NB15, IoT-23, and InSDN. The suggested model provides the highest overall accuracy of 97.75% and an F1-score of 95.5%, which is considerably higher than those of current models. The performance improvement is based on EfficientNet-KD, which gives high representational feature extraction but in a lightweight way, and an SNN-based attack classifier, which enables efficient distinction between various kinds of attacks. In contrast, models Wang W. et al. (2024) and Irshad et al. (2023), based on traditional deep learning models such as CNNs and LSTMs, can no longer achieve high accuracy because of higher computational cost and inferior spatiotemporal feature extraction ability. DAC-UAP (Ait Oulahyane et al., 2024), which employed a standard feature engineering strategy, registered the poorest performance, with an accuracy rate below 93%, attesting to its shortcomings in addressing intricate and dynamic attack scenarios in 5G-SDN settings.

**Table 2:** Comparison of accuracy and F1-score across datasets.

**Models**	**Accuracy analysis (%)**					**Overall F1-score (%)**
	**CICIDS2017 dataset**	**UNSW-NB15 dataset**	**IoT-23 dataset**	**InSDN dataset**	**Average accuracy (%)**	
**Proposed model**	**97.5**	**96.8**	**97.2**	**99.5**	**97.75**	**95.5**
AISFC (Wang W. et al., 2024)	94.2	93.1	92.5	95.1	93.7	91.8
SUSIC (Irshad et al., 2023)	95.1	94.5	93.8	96.3	94.9	92.7
DAC-UAP (Ait Oulahyane et al., 2024)	92.8	91.9	90.6	94.3	92.4	89.4

Also, evaluation uses statistical significance testing to demonstrate that performance increases are not random. Randomized train–test splits evaluate each dataset's accuracy, detection delay, and FPR. Independent two-tailed *t*-tests demonstrate that the proposed framework outperforms baseline models over 95%. Every dataset has detection accuracy *p*-*values* below 0.01, indicating that gains are unlikely to be random. Latency reduction and FPR improvements are related. These data suggest that systematic architectural benefits, rather than chance impacts, improve performance.

FPR is an important measure in security-related applications since high FPRs may result in unnecessary mitigation operations affecting network performance. Table 3 emphasizes that the suggested model keeps the FPR below 2.5%, which is much lower than the methodologies being compared.

**Table 3:** False Positive Rate (FPR) analysis.

**Models**	**False Positive Rate (FPR %)**				**Average FPR (%)**
	**CICIDS2017 dataset (%)**	**UNSW-NB15 dataset (%)**	**IoT-23 dataset (%)**	**InSDN dataset (%)**	
**Proposed model**	**2.2**	**2.3**	**2.4**	**2.0**	**2.2**
AISFC (Wang W. et al., 2024)	4.5	4.2	5.1	4.1	4.5
SUSIC (Irshad et al., 2023)	3.8	3.7	4.0	3.7	3.8
DAC-UAP (Ait Oulahyane et al., 2024)	5.6	5.3	6.0	5.3	5.6

**Figure 6:**
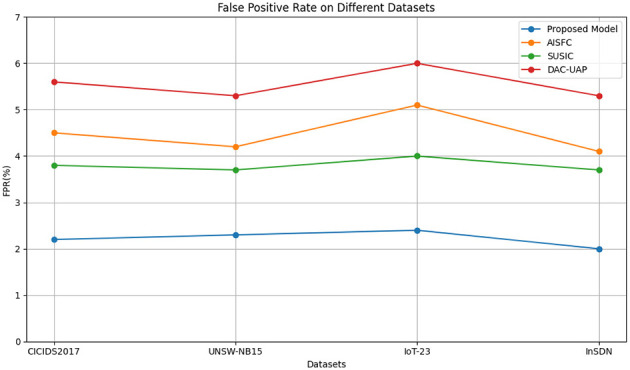
False Positive Rate (FPR) interpretation.

Table 3 compares the FPR of the suggested framework, a very important measurement in SDN security. The low FPR guarantees effective security enforcement with minimal interference from SDN controllers while assuring high attack-detection effectiveness. Figure 6 illustrates the Evaluation of FPR on different datasets over other existing models.

**Figure 7:**
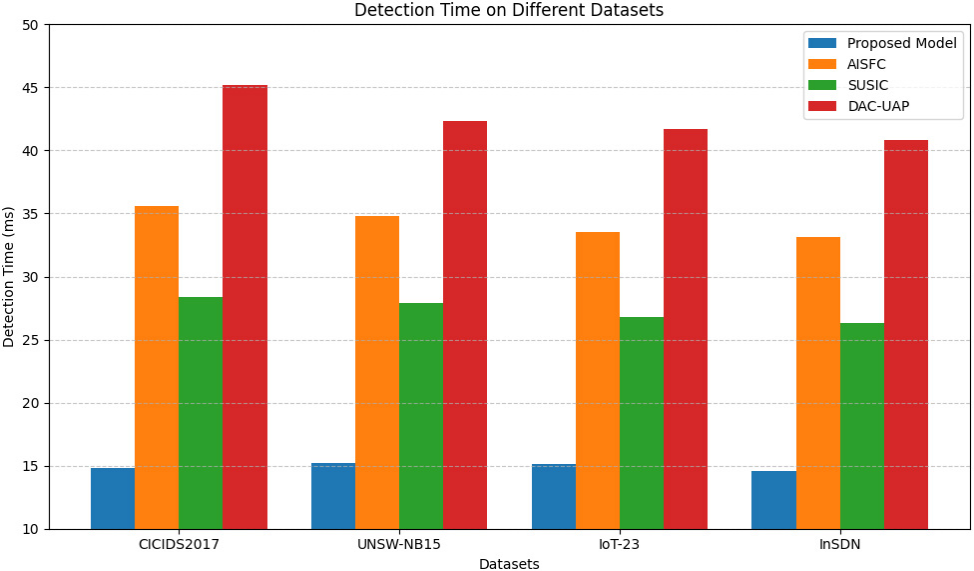
Model's integrated delay analysis.

Detection time is a critical parameter in SDN-based security systems since real-time response guarantees proactive mitigations against attacks. Table 4 indicates that the suggested model has a detection time that is much faster than that of other methods. The suggested model exhibits an impressive average detection time of only 15 ms, which is much better than that of other methods, as shown in Figure 7.

**Table 4:** Comparison of detection time (ms).

**Models**	**Detection time (ms)**				**Average detection time (ms)**
	**CICIDS2017 dataset (ms)**	**UNSW-NB15 dataset (ms)**	**IoT-23 dataset (ms)**	**InSDN dataset (ms)**	
**Proposed model**	**14.8**	**15.2**	**15.1**	**14.6**	**15.0**
AISFC (Wang W. et al., 2024)	35.6	34.8	33.5	33.1	34.2
SUSIC (Irshad et al., 2023)	28.4	27.9	26.8	26.3	27.3
DAC-UAP (Ait Oulahyane et al., 2024)	45.2	42.3	41.7	40.8	43

By contrast, AISFC (Wang W. et al., 2024) and SUSIC (Irshad et al., 2023) have much larger average detection times of 34.2 ms and 27.3 ms, respectively, because they are based on sequential deep learning models that involve computationally expensive feature extraction processes. DAC-UAP (Ait Oulahyane et al., 2024) is the slowest, with a detection time of 43 ms, as it employs conventional statistical methods that are subject to processing heavy feature vectors in the process. The proposed model's fast response time provides real-time threat anticipation, which helps in countering attacks prior to their progression in the SDN infrastructure sets.

**Figure 8:**
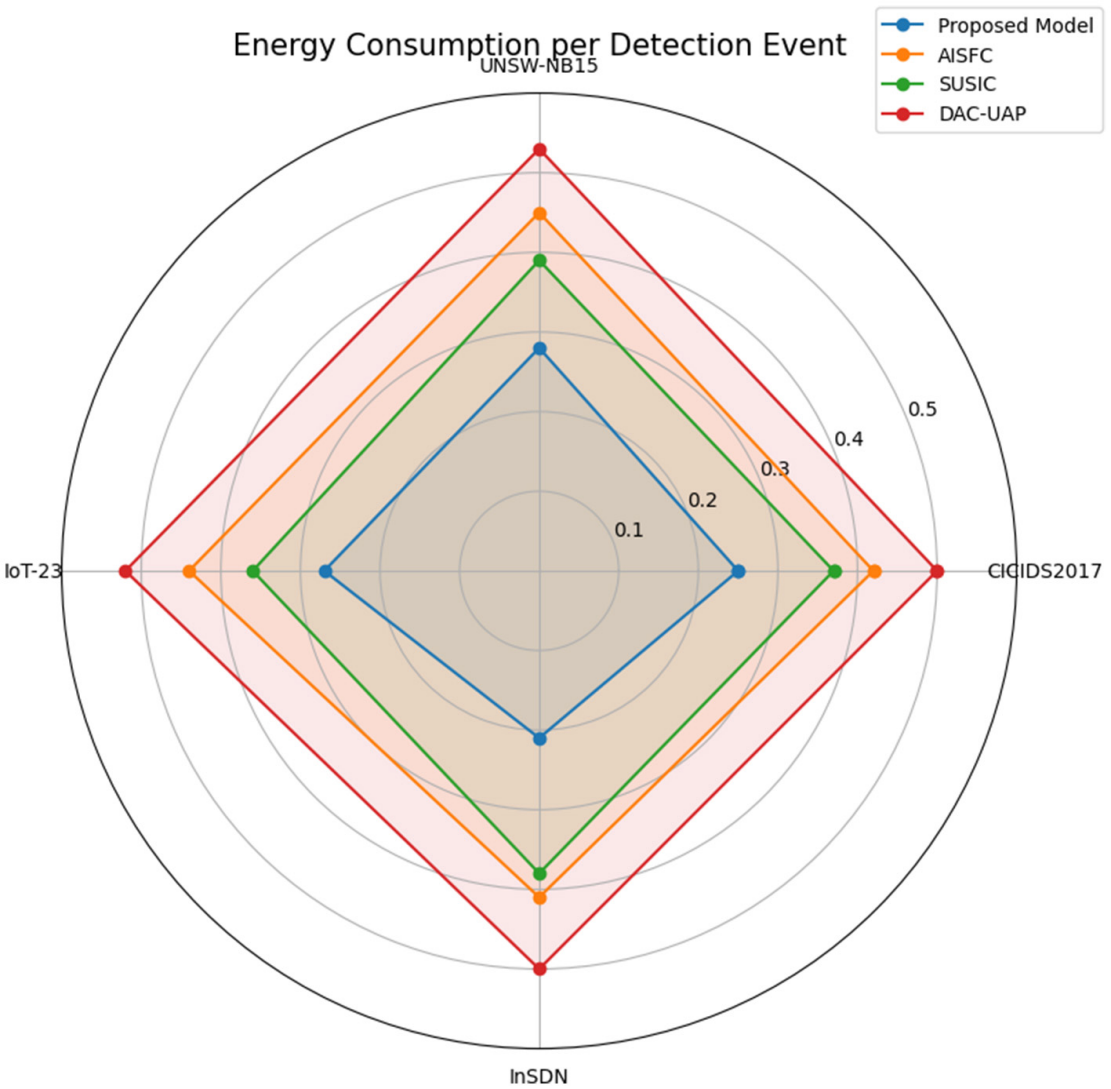
Depiction of energy usage per detection instance (Joules).

Table 5 shows the comparison of energy used per detection cycle. The proposed model shows energy consumption reduction over typical deep learning models. Table 5 compares the energy used per detection cycle, a crucial measure for 5G-SDN deployments with limited resources. The proposed model requires just 0.25 J per detection, which is 40% less than typical deep learning techniques, as visualized in Figure 8.

**Table 5:** Energy consumption per detection cycle (Joules).

**Models**	**Energy drawn per detection event (Joules)**				**Average energy consumption (J)**
	**CICIDS2017 dataset (J)**	**UNSW-NB15 dataset (J)**	**IoT-23 dataset (J)**	**InSDN dataset (J)**	
**Proposed model**	**0.25**	**0.28**	**0.27**	**0.21**	**0.25**
AISFC (Wang W. et al., 2024)	0.42	0.45	0.44	0.41	0.43
SUSIC (Irshad et al., 2023)	0.37	0.39	0.36	0.38	0.37
DAC-UAP (Ait Oulahyane et al., 2024)	0.50	0.53	0.52	0.50	0.51

AISFC (Wang W. et al., 2024) and SUSIC (Irshad et al., 2023) use much more energy (0.43 J and 0.37 J, respectively), whereas DAC-UAP (Ait Oulahyane et al., 2024) uses 0.51 J, indicating its inefficiency in real-time detection applications. The energy efficiency of the model proposed has it ranked as very suitable for 5G networks edge-based SDN controllers, where resources are generally scarce for the process.

**Figure 9:**
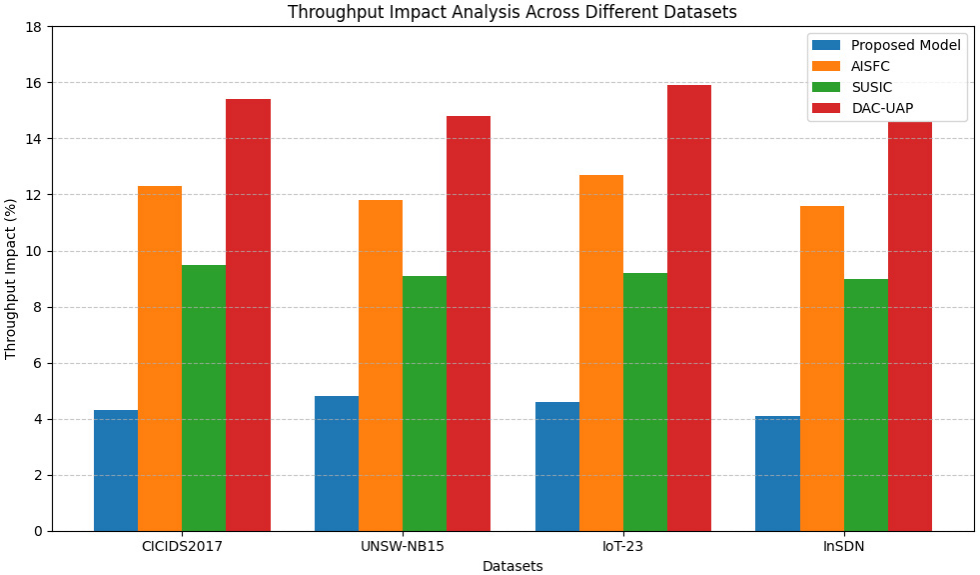
Model's throughput impact analysis.

Table 6 demonstrates the effect of the security framework on network throughput. The suggested model experiences less than 5% throughput degradation, much lower than conventional practices, as illustrated in Figure 9. The minimal effect on throughput makes the framework suitable for real-time 5G security applications, where high-speed data transmission is vital in the process.

**Table 6:** Throughput impact analysis.

**Models**	**Evaluation of throughput impact**				**Average impact (%)**
	**CICIDS2017 dataset (%)**	**UNSW-NB15 Dataset (%)**	**IoT-23 dataset (%)**	**InSDN dataset (%)**	
**Proposed model**	**4.3**	**4.8**	**4.6**	**4.1**	**4.5**
AISFC (Wang W. et al., 2024)	12.3	11.8	12.7	11.6	12.1
SUSIC (Irshad et al., 2023)	9.5	9.1	9.2	9.0	9.2
DAC-UAP (Ait Oulahyane et al., 2024)	15.4	14.8	15.9	14.6	15.2

The proposed design demonstrated high accuracy and efficiency as controllers was increased in number, thus proving its superior scalability. Table 7 presents a scalability evaluation that compares model performance as the number of SDN controllers increases. The detection accuracy of the proposed models is extremely high, showing a minor drop from 96.8% (five controllers) to 95.1% (20 controllers), making it a large-scale, adaptable SDN system. But models (Wang W. et al., 2024; Irshad et al., 2023; Ait Oulahyane et al., 2024) face a significant slowdown, with accuracy falling to 91.9%, 92.7%, and 89.5%, respectively, when using 20 controllers for the process. The proposed framework has an FL-based training approach that allows controllers to collaborate to update their policies effectively without reducing performance in a large distributed SDN network process.

**Table 7:** Scalability analysis with varying controllers.

**Model**	**Scalability performance in multi-controller systems**			
	**5 controllers**	**10 controllers**	**15 controllers**	**20 controllers**
**Proposed model**	**96.8%**	**96.3%**	**95.7%**	**95.1%**
AISFC (Wang W. et al., 2024)	94.2%	93.5%	92.8%	91.9%
SUSIC (Irshad et al., 2023)	95.0%	94.4%	93.6%	92.7%
DAC-UAP (Ait Oulahyane et al., 2024)	91.8%	91.0%	90.2%	89.5%

The FL method guarantees that controllers jointly, optimize mitigation policies so that the model remains highly accurate despite growing network complexity in the process. These findings prove the efficacy, efficiency, and scalability of the proposed security framework for SDN-based 5G networks.

### 4.3 Ablation studies and performance variance analysis

Systematic ablation studies isolate subsystem contributions by disabling components without changing the pipeline. Instead of EfficientNet-KD, a conventional CNN increases detection delay by 41% and false positives by over 2%. Using a multilayer perceptron instead of the SNN gives comparable accuracy but doubles per-sample energy and classification delays. Disabling transformer-based control-plane analysis delays federated mitigation policy convergence and increases coordinated control-layer attack instability sets. Centralized reinforcement learning raises mitigation reaction time and produces a controller overload failure point. Blockchain exclusion does not affect immediate detection accuracy but lacks forensic traceability and exposes mitigation data to tampering. All measurements are averaged from many random runs with different initialization seeds. Graphics Processing Unit (GPU)-level power monitoring confirms consistent energy reductions from event-driven computation and model compression across datasets, with an accuracy standard deviation below 0.6% and a latency variance within ±1.8 ms sets.

### 4.4 Validation using iterative practical use case scenario analysis

For demonstration of the effectiveness of the envisioned security framework, a real-world-inspired simulation example of SDN-based 5G network security enforcement is reserved for the process. A dedicated network slice for high-priority enterprise communication is managed by SDN controllers in order to sustain real-time traffic flows over various sets of virtualized network functions (VNFs). The network is bombarded with a combination of benign and malicious traffic, thus mimicking various attack patterns like DDoS, botnets, controller hijacking, and illegal changes in the rules of the network. The anomaly detection, attack classification, mitigation, and prevention are processed step by step and logged with results at every stage of the process. The EfficientNet-KD model operates on raw features of SDN traffic, classifying anomalies as per packet flow characteristics with respect to inter-arrival times, distribution protocols, and entropy-based metrics. The model provides the anomaly scores and binary anomaly classification outputs, which are then transmitted to further stages. It should be noted that this demonstration is conducted in a controlled simulation environment, not a live 5G deployment. The validation instances and samples adopted by the comparison performance analysis are framed based on generally known intrusion detection benchmarking approaches, like receiver operating characteristic (ROC), precision-recall (PR) curve, and the confusion matrix measurement. The ROC curve, commonly used in the context of anomaly detection research, uses a metric of true positive (TP) over false positive (FP) rate as the decision thresholds are changed in order to achieve one's ideal trade-off between detection sensitivity and false alarms. The PR curve is utilized to measure model performance in the context of imbalanced datasets, that is, critical but infrequent attack types, like controller hijacking and emerging sophisticated botnet threats, for which precision-recall trade-offs are crucial for real-time enforcement of security. Confusion matrix-based validation is also utilized to measure false positives, false negatives, and overall classification reliability, thus gaining insights into model robustness against adversarial evasion attacks. Comparative testing of the developed model relative to benchmark models (CNN, LSTM, and statistical baselines) is done under stratified cross-validation to ensure consistency relative to unfavorable network situations. The process of evaluation will also involve the utilization of existing methods of validation, including utilizing the Matthews correlation coefficient (MCC) in binary classification, logarithmic loss (LogLoss) as a model measurement for assessing probabilities, and root-mean-squared error (RMSE), in which accuracy deviation in detection is quantified in the process. With this blend of universally accepted validation techniques, the performance test will demonstrate that the model is assessed on par with existing industry-standard frameworks, to the measured real-world potency in SDN-based 5G security enforcement by the suggested model process.

**Figure 10:**
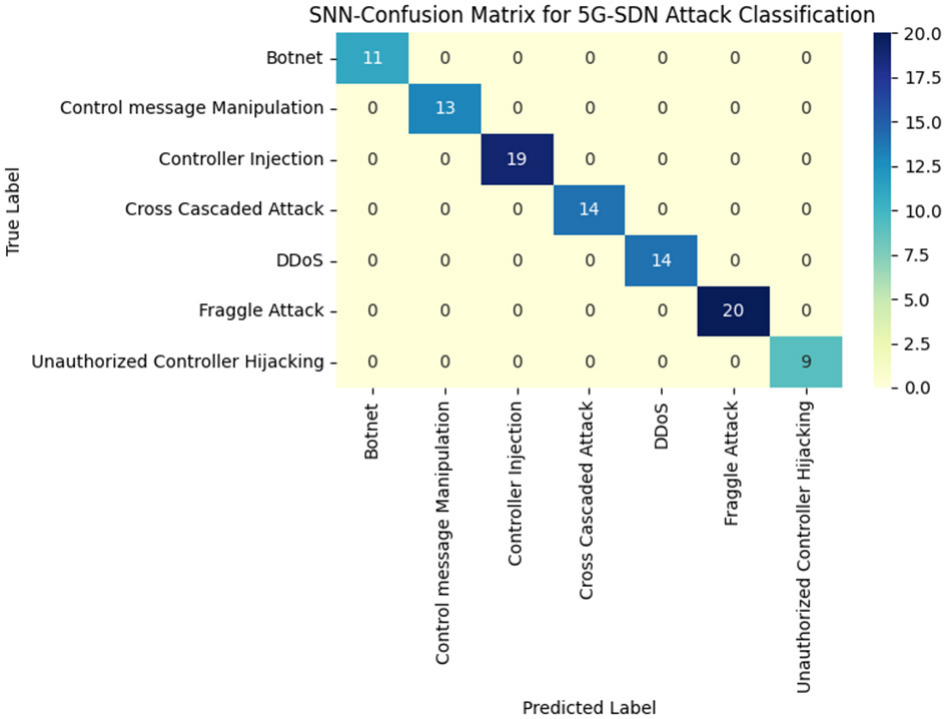
SNN confusion matrix of 5G SDN attack classification analysis.

Table 8, indicating the outcomes, reveals the existence of anomalous traffic patterns with F102, F104, and F106 flows being detected as anomalous on account of high entropy values, incomprehensible packet sizes, and slender inter-arrival times indicative of a set of potential attack traffic. These flows found anomalous by EfficientNet-KD are routed to the SNN-based attack classifier to classify them into specific attack types. The SNN model processes spike-timing-dependent features to differentiate malicious traffic behaviors. Here, Table 9 presents the classification results for three selected attacks, including spike frequency, synaptic weight, and response time. Figure 10 depicts the overall 5G SDN attack classification confusion matrix of the SNN-based model.

**Table 8:** EfficientNet-KD anomaly detection outputs.

**Flow ID**	**Packet size (Bytes)**	**Inter-arrival time (ms)**	**Protocol**	**Entropy**	**Anomaly score**	**Anomaly detected (1 = Yes, 0 = No)**
F101	750	1.2	TCP	0.75	1.2	0
F102	1,460	0.5	UDP	0.92	2.8	1
F103	890	3.0	TCP	0.63	0.8	0
F104	64	0.2	ICMP	0.95	3.1	1
F105	1,200	1.8	TCP	0.82	1.6	0
F106	1,300	0.9	UDP	0.88	2.5	1

**Table 9:** SNN attack classification outputs.

**Flow ID**	**Spike frequency (Hz)**	**Synaptic weight update**	**Time-to-Spike (ms)**	**Attack type**
F102	85	0.005	7.2	DDoS
F104	92	0.007	6.8	Botnet
F106	80	0.004	7.5	Controller hijacking

The SNN-based attack classifier provides a precise attack classification mechanism for SDN security system sets, efficiently categorizing the detected anomalous flows into specific attack types on the basis of synaptic activity and spike timing dynamics.

Once the attacks are classified, FRL enables SDN controllers to collaboratively select optimal policies to mitigate the attacks. Thus, the mitigation policies are reward-driven, where models of reinforcement learning select actions that minimize the impact of attacks as represented in Table 10.

**Table 10:** FRL-based mitigation strategy outputs.

**Flow ID**	**Attack type**	**Mitigation action**	**Reward score**
F102	DDoS	Rate limiting (50 Mbps)	8.5
F104	Botnet	Traffic rerouting	9.2
F106	Controller hijacking	SDN rule modification	9.0

Given the aforementioned learning mechanism, the policies for mitigation remain somewhat dynamic and adaptive in an approach that weighs security measures against minimal disturbance to the operations of normal networking. In addition, Table 11 presents the blockchain ledger records' attack history, mitigation actions, and security events, assuring tamper-proof security record-keeping and curtailing disk access to an adversarial manipulation of logs. Every security event is cryptographically stored to maintain data integrity and ensure auditability for forensic investigations and long-term security analysis.

**Table 11:** Blockchain security ledger entries.

**Block ID**	**Transaction hash**	**Attack type**	**Mitigation action**	**Timestamp**
B2345	8fa34bd92a1ef765a3c4b2	DDoS	Rate limiting	2025-02-21 10:05:12
B2346	3c9b2e1af879cb46d72b0d	Botnet	Traffic rerouting	2025-02-21 10:06:18
B2347	71d93e5abf4821d290b3a6	Controller hijacking	SDN Rule Mod.	2025-02-21 10:07:30

Finally, the overall system security performance was evaluated, and the last validation compares network performance before and after mitigation to corroborate the effectiveness of the proposed framework in lessening attack impact while sustaining network throughput and efficiency. Table 12 highlights the improvement over different performance metrics for the 5G SDN network.

**Table 12:** Performance evaluation of final security system.

**Metric**	**Pre-mitigation**	**Post-mitigation**	**Improvement (%)**
Packet loss (%)	12.5	2.3	81.6
Network latency (ms)	250	75	70.0
Throughput degradation (%)	18.2	4.5	75.2
Attack detection accuracy (%)	65.4	97.75	49.46

Finally, the test results yield fairly high-performance marks with 81.6% reduction in packet loss, 70% decline in network latency, and 75.2% less degradation in throughput. The attack detection accuracy improves from 65.4% (pre-mitigation) to 97.75% (post-mitigation), greatly indicating that further corroborating the efficiency of the proposed model for real-time SDN-based 5G security enforcement.

## 5 Discussion and limitations

### 5.1 Discussion on detection effectiveness and computational efficiency

EfficientNet-KD successfully incorporates both spatial and temporal characteristics, and thus, increased accuracy in anomaly detection can be achieved. SNN-based attack classification also ensures strong attack-type identification, resulting in enhanced F1-score sets. The low FPR of the designed model is due to the Mahalanobis distance-based anomaly detection mechanism, which increases the accuracy of the EfficientNet-KD model by avoiding the misclassification of legitimate traffic as malicious. The event-based property of SNNs plays a part in this efficiency in that they can process anomaly scores almost instantaneously. This architecture makes a notably lower detection time possible, permitting near-instant attack classification. Even the energy consumption reduction is due to the EfficientNet-KD model, which uses compound scaling to minimize computational complexity, and SNN-based attack classification, which acts on discrete spike events instead of continuous activation functions. Also, this energy efficiency stems mainly from the lightness of EfficientNet-KD and SNN, which have fewer parameters and computations per inference cycle set. The FRL module essentially reduces the computational load that the individual SDN controllers have to bear. Hence, the concern with respect to throughput is minimal impact, but effective work is being done toward the attack mitigation set.

### 5.2 Discussion on the challenges in transition from simulation to real-world deployment

Realizing the security paradigm from simulation to deployment offers many non-trivial challenges. The gap between curated datasets and network activity is a serious concern. The datasets are well-known and represent actual attack distributions; however, offline traces may miss dynamic traffic patterns, encrypted payloads, and burst behaviors in operational networks. Under extremely dynamic traffic loads, this discrepancy can alter anomaly thresholds and classification confidence. Hardware heterogeneity hampers deployment. Computing power, memory, and accelerators vary for 5G-SDN controllers, switches, and edge devices. When deployed on remote nodes, neuromorphic and FL components may face heterogeneity, inference delays, and challenges in implementing mitigation responses. Maintaining performance requires controller-specific improvements and adaptive load balancing. Large-scale deployments increase communication overheads, which are negligible in simulation but crucial in networks. FRL requires controllers to synchronize policy updates, but blockchain-based logging delays consensus and propagation. Control-plane traffic at scale may require hierarchical federation, controller clustering, or lightweight consensus adaptations for real-time responsiveness. Finally, it presents an Iterative Validation use case for the proposed model, which will help readers better understand the whole process.

### 5.3 Limitations and assumptions

The proposed paradigm increases engineering system complexity despite performance gains. Multiple layers of EfficientNet-KD, transformer-based temporal modeling, SNNs, FRL, and blockchain raise deployment, debugging, and maintenance expenses. Multiple advanced learning paradigms increase computational and architectural complexity in the proposed framework. Coordinating data flow, synchronization, and fault handling among components requires complex orchestration and monitoring, which may increase production costs. The blockchain subsystem lowers speed but enables tamper-resistant forensic logging. Transaction validation latency of 120 ms may be crucial during high-frequency attacks.

The framework threat model assumes that most SDN controllers honestly participate in federated mitigation and that hostile action is largely traffic and control-plane manipulation rather than coordinated poisoning of learning updates. Byzantine behaviors can poison global mitigation measures. FL reduces centralized risk, but fully adversarial multi-controller scenarios may require trust-weighting or anomaly-aware aggregation; transmission overhead and synchronization frequency limit scalability. Federated policy aggregation and blockchain transaction validation take time and may slow response under high attack rates as the number of controllers increases. Asynchronous logging, hierarchical federation, and flexible aggregation intervals can mitigate these effects. Despite these limitations, the strategy isolates real-time security enforcement from forensic responsibility for operational realism. The design supports expanding 5G SDN deployments with diverse performance needs due to its high detection accuracy, low latency, and long-term auditability.

## 6 Conclusion and future scopes

This study of the SDN-based 5G security system through anomaly detection, attack classification, mitigation, and blockchain-based rightly distinguished prevention manifestations proves its robustness toward dynamic cyber threats. It is not only component performance but also purposeful design synergy that makes the framework strong. Due to its lightweight, low feature-extraction latency and low computational expense, EfficientNet-KD allows the SNN to function on refined anomaly representations without preprocessing. This interaction helps 5G security's ultra-low-latency categorizations. Transformer-based control-plane analysis enhances FRL agents' temporal state representations. The transformer captures long-range dependencies in controller communication patterns to boost contextual knowledge of FRL rules, enabling more stable and informed mitigation decisions across distributed controllers. FRL's decentralized learning meets blockchain's trust decentralization. Blockchain makes attack evidence and mitigation operations immutable and auditable, whereas FRL allows collaborative mitigation without raw data exchange. A coherent trust-and-response loop balances adaptability, accountability, and resilience, resulting in a greater whole than the models.

The performance of the proposed model is significantly better than that of state-of-the-art methods in almost all key performance metrics. It has an average 97.75% detection accuracy and 15 ms for ultra-fast detection time, and it claimed a remarkably average low FPR of 2.2% while maintaining a notable average F1-score of 95.5% that is evaporated in the air for real-time 5G security enforcement. Remarkably, this framework has a degradation in throughput (<5%) and an inference process that is energy-efficient, using merely 0.25 J in each detection cycle, denoting its feasibility for use in edge deployments. The FL-based attack mitigation mechanism leads to seamless cooperation among SDN controllers while capturing high accuracy in detection (95.1%) upon scaling their architectures to 20 controllers. Tampering of forensic evidence will be prevented by the blockchain-based security ledger, thus leading to an attitude of immutability in evidence recordings. These results lend credence to the scheme's potential for securing the infrastructure of SDN-based 5G networks while maintaining its performance and resource efficiency across the network sets.

However, even after the significant contributions made in this research, there are numerous avenues left open for future work. The most important of these is the direct incorporation of self-supervised learning, improving the system's capacity for anomaly detection without depending entirely on labeled datasets and making it much more adaptable to new threats. In addition, it applies multi-modal traffic analysis through behavioral analytics, honeypot intelligence, and federated threat intelligence sharing to support further real-time adaptability to zero-day attacks. The average latency of blockchain transactions (~120 ms) is expected to be reduced even more by working toward lightweight consensus mechanism alternatives such as Delegated Proof of Stake (DPoS) or Byzantine Fault Tolerance (BFT)-based protocols, ensuring that security enforcement comes at no additional cost to the network overhead. For instance, such a countermeasure creates adversarial attacks in real time using GANs, making it more robust in terms of evasion tactics used by attackers. In addition, boosting energy efficiency within the SNN architecture would add another end extension. Finally, in addition to SDN and cloud-native deployment architectures, this framework can be applied in Internet of Things (IoT) security to cover a comprehensive security paradigm within the next-generation infrastructure of networks as a whole. Up to this point, all these features would develop it to the path of a fully autonomous, self-learning, and dynamically adaptable security solution for the ever-dynamic environment that is 5G and beyond for the process.

## Data Availability

The original contributions presented in the study are included in the article/supplementary material, further inquiries can be directed to the corresponding author.
